# Pick a pattern: Transcriptional regulator allelic diversity synergistically drives trichome pattern diversity

**DOI:** 10.1093/plphys/kiaf058

**Published:** 2025-03-04

**Authors:** Lara Pereira, Erin Cullen

**Affiliations:** Assistant Features Editor, Plant Physiology, American Society of Plant Biologists; Ecology and Evolutionary Biology, School of Biosciences, University of Sheffield, Sheffield S10 2TN, UK; Assistant Features Editor, Plant Physiology, American Society of Plant Biologists

Trichomes, commonly referred to as plant hairs, are highly specialized from aerial epidermal cells. These structures can be morphologically very diverse: uni- or multicellular, branched or not branched, and glandular or nonglandular. Their ecological functions are also diverse, with predominant roles in plant defense against biotic and abiotic stresses. For example, trichomes can provide a physical and chemical barrier to herbivores, protect against UV damage by absorbing harmful radiation, and buffer against drought stress (Fürstenberg-Hägg et al. 2013; Bickford 2016).

In the model species *Arabidopsis thaliana*, an intricate network of transcriptional regulators controlling trichome development has been elucidated (Yang and Ye 2013). A trimeric MYB/bHLH/WD-repeat complex, composed of GLABRA1 (GL1), GLABRA3/ENHANCER OF GLABRA2 (GL3/EGL3), and TRANSPARENT TESTA GLABRA1 (TTG1), activates the expression of the homeodomain transcription factor GLABRA2 (GL2), which triggers trichome fate in epidermal cells (Fig.). In addition, another 7 MYB transcription factors act as negative regulators by interfering with the trimeric activation complex.

**Figure.**
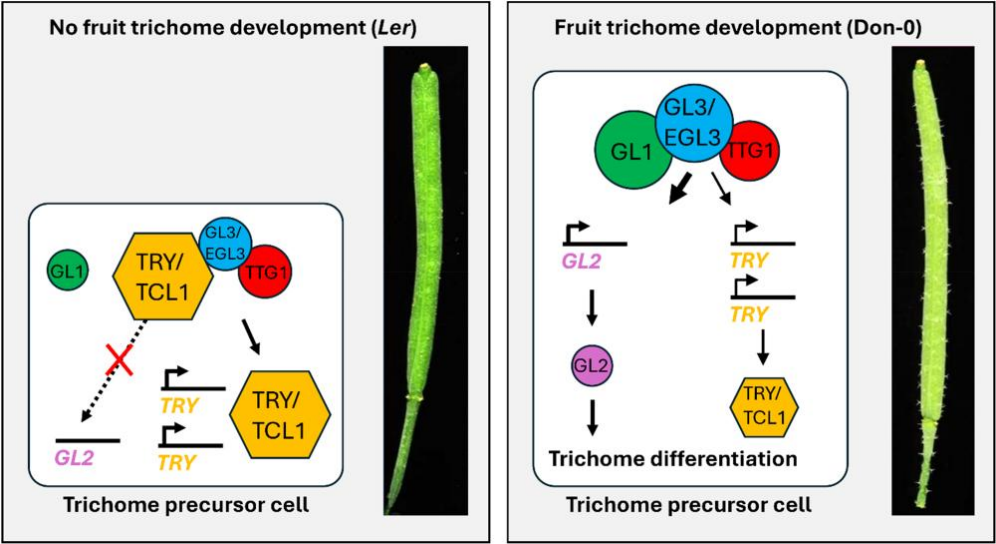
Model of the molecular mechanisms underpinning trichome diversity in Arabidopsis, leading to the glabrous fruits of the *Ler* accession and hairy fruits of the Don-0 accession. Transcription factors in the core gene regulatory network known to be involved in trichome patterning from this study (*EGL3*) and previous work are illustrated (*GL1*, *TRY*, and *TCL1*) (Arteaga et al. 2021). Wider transcriptional arrows and larger protein symbols indicate higher activity (*EGL3*, *GL1*), or larger amounts (*TRY* and *TCL1*). Schematic adapted from Figure 6, Méndez-Vigo et al. (2024).

Trichomes can appear in all aerial parts of the plant, and the quantity and distribution, or trichome patterning, is diverse in plants. The inter- and intraspecific variation in trichome patterning is genetically defined as an adaptive response to their local environments, although the trait is also highly plastic (Arteaga et al. 2022). Trichome patterning often differs between populations of the same species and is theorized to be a trade-off between increased fitness in challenging environments and the cost of trichome production (Hauser 2014). In Arabidopsis, leaf trichome density is linked to a lack of precipitation in spring (Arteaga et al. 2022), whereas in the related Brassicaceae species *Cardamine hirsuta*, high leaf trichome density is associated with high spring precipitation (Fuster-Pons et al. 2024). Therefore, trichome density appears to vary in different species in response to specific environmental pressures.

Classically, Arabidopsis has been taxonomically characterized by the absence of trichomes in fruits. However, the Doñana (Don-0) accession develops trichomes in fruits and pedicels, representing an evolutionary innovation likely involved in local adaptation. Don-0 belongs to an ancient relict lineage from the Iberian Peninsula, a genetic group that diverged from nonrelict accessions before the last glaciation (Alonso-Blanco et al. 2016). A mapping population developed to genetically dissect this natural variation revealed 5 quantitative trait loci (QTL) involved in trichome patterning. Cloning of the 3 major QTLs found that fruit trichome development in Don-0 is due to loss of function in the trichome repressors TRICHOMELESS1 (TCL1) and TRIPTYCHON (TRY) and gain-of function in the trichome activator GL1 (Arteaga et al. 2021).

In a recent paper published in *Plant Physiology*, Méndez-Vigo et al. (2024) used comprehensive genetic and molecular analyses, in combination with phylogenetic studies, to characterize the QTL *MALAMBRUNO 1* (*MAU1*), which was previously found to be involved in trichome patterning in the Don-0 accession (Arteaga et al. 2021). First, a set of introgression lines with all the possible allelic combinations from Don-0 and the laboratory strain Landsberg *erecta* (L*er*) was developed. These lines were phenotyped for trichome traits to characterize the effects of *MAU1* and the interactions with other genetic factors controlling trichome patterning. The authors showed that the Don-0 *MAU1* allele plays a key role in fruit trichome appearance, but only when Don-0 alleles are present on other QTLs. In fact, the partial loss-of-function Don-0 allele at the negative regulator TRY is required to develop a high number of fruit trichomes.

Fine mapping of *MAU1* revealed that the underlying gene is *EGL3* (Fig.), which encodes a bHLH transcription factor previously shown to regulate trichome pattern in Arabidopsis leaves (Zhang et al. 2003). The authors tested the functionality of Don-0 and *Ler* alleles by introducing genomic constructs with the promoter, coding and 3'-UTR regions in the double mutant background and introgression lines. They showed that Don-0 carries a gain-of-function allele of *EGL3* acting specifically on fruits. The gene expression of *EGL3* in the transgenic lines was not significantly different; therefore, the phenotypic effects are most likely caused by differences in protein structure and function rather than *cis*-regulatory mutations. Instead, one missense mutation, causing an amino acid change from Asn to Ser at position 12 within the MYC N-terminal protein domain, seems to be responsible for changes in trichome patterning in Don-0.

To uncover the role of *EGL3* in the natural variation of trichome patterning, a phylogenetic analysis was performed. The nucleotide sequences of 235 Iberian accessions were compared, utilizing published genome sequences (Alonso-Blanco et al. 2016). Two major haplogroups were described, and most polymorphisms affected the promoter and exons 5 and 6. However, analysis of *EGL3* in 23 Brassicaceae species revealed that these main haplogroups likely originated earlier in Arabidopsis history and are not associated with trichome pattern variation. The authors then focused on the 21 Iberian accessions that develop trichomes in fruits. Three distinct subhaplogroups carrying different low-frequency polymorphisms at the *EGL3* gene were observed, suggesting that several mutations may affect the phenotype. Interestingly, multiple independent gain-of-function alleles were identified. Furthermore, these haplotypes were restricted to accessions from the Iberian Peninsula, indicating that these specific alleles originated in this region likely as a mechanism to climatic adaptation (Arteaga et al. 2022).

This study shines light on the genetic basis of trichome pattern diversity in the Arabidopsis relict lineage. The authors elegantly show that synergistic interactions between the natural gain-of-function *EGL3* allele and specific alleles of other regulators, also involved in leaf trichome initiation, underlie Arabidopsis fruit trichome development in the Don-0 accession. Arteaga et al. (2022) found that Arabidopsis leaf trichomes may be linked to low precipitation, and therefore Arabidopsis fruit trichomes may also be correlated with this climatic variable. Consequently, it would be interesting to test the functional relevance of Arabidopsis fruit trichomes. For example, do fruit trichomes increase resilience to abiotic stressors, such as drought, or biotic stressors, such as herbivores? Ultimately, research about the role of *EGL3* in trichome patterning in crop species such as tomato and cotton could be used in plant breeding to boost crop resilience.

## Data Availability

No data were generated or analyzed in this study.
